# A novel point mutation in a class IV glucose-6-phosphate dehydrogenase variant (*G6PD São Paulo*) and polymorphic *G6PD* variants in São Paulo State, Brazil

**DOI:** 10.1590/S1415-47572009005000033

**Published:** 2009-06-01

**Authors:** Raimundo Antonio G. Oliveira, Marilena Oshiro, Mario H. Hirata, Rosario D. C. Hirata, Georgina S. Ribeiro, Tereza M. D. Medeiros, Orlando C. de O. Barretto

**Affiliations:** 1 Departamento de Farmácia, Universidade Federal do Maranhão, São Luis, MABrazil; 2 Instituto Adolfo Lutz, São Paulo, SPBrazil; 3 Departamento de Análises Clínicas e Toxicológicas, Faculdade de Ciências Farmacêuticas, Universidade de São Paulo, São Paulo, SPBrazil; 4 Departamento de Patologia e Apoio Clínico, Universidade Federal Fluminense, Niterói, RJBrazil; 5 Departamento de Análises Clínicas e Toxicológicas, Universidade Federal do Rio Grande do Norte, Natal, RNBrazil; 6 Laboratório LIM-23, Faculdade de Medicina, Universidade de São Paulo, São Paulo, SPBrazil

**Keywords:** glucose-6-phosphate dehydrogenase, mutations, polymorphism, variants

## Abstract

In this study, we used red cell glucose-6-phosphate dehydrogenase (G6PD) activity to screen for G6PD-deficient individuals in 373 unrelated asymptomatic adult men who were working with insecticides (organophosphorus and carbamate) in dengue prevention programs in 27 cities in São Paulo State, Brazil. Twenty-one unrelated male children suspected of having erythroenzymopathy who were attended at hospitals in São Paulo city were also studied. Fifteen of the 373 adults and 12 of the 21 children were G6PD deficient. *G6PD* gene mutations were investigated in these *G6PD*-deficient individuals by using PCR-RFLP, PCR-SSCP analysis and DNA sequencing. Twelve *G6PD* A-202A/376G and two *G6PD* Seattle844C, as well as a new variant identified as *G6PD* São Paulo, were detected among adults, and 11 *G6PD* A-202A/376G and one *G6PD* Seattle844C were found among children. The novel mutation c.660C > G caused the replacement of isoleucine by methionine (I220M) in a region near the dimer interface of the molecule. The conservative nature of this mutation (substitution of a nonpolar aliphatic amino acid for another one) could explain why there was no corresponding change in the loss of G6PD activity (64.5% of normal activity in both cases).

Over 440 glucose-6-phosphate dehydrogenase (G6PD) variants (Beutler and Vulliamy, 2002) have been grouped in five classes according to their enzymatic activity and clinical manifestations (World Health Organization-WHO criteria) (Beutler and Yoshida, 1988). The *G6PD* gene is highly polymorphic and so far more than 150 mutations resulting in distinct G6PD variants have been described (Beutler and Vulliamy, 2002; Hamel *et al.*, 2002; Rodrigues *et al.*, 2002; Vaca *et al.*, 2003; Drousiotou *et al.*, 2004; Grabowska *et al.*, 2004; van Wijk *et al.*, 2004; Yan *et al.*, 2006; Maciag *et al.*, 2007; Mason *et al.*, 2007; Matsuoka *et al.*, 2007; McDade *et al.*, 2008; Minucci *et al.*, 2008; Nuchprayoon *et al.*, 2008). Most of these mutations show low enzyme activity and only five are class IV variants: *G6PD* São Borja337G > A (Weimer *et al.*, 1993), *G6PD* A376A > G (Takizawa *et al.*, 1987), *G6PD* San Luis Potosi376A > T (Vaca *et al.*, 2003), *G6PD* Insuli989G > A (Sukumar *et al.*, 2003), and *G6PD* Mira d'Aire1048G > C (Beutler and Vulliamy, 2002).

In this study, we screened for *G6PD* gene mutations in a series of G6PD-deficient adult males who were working with insecticides (organophosphorus and carbamate) in dengue prevention and in a group of unrelated male children suspected of having erythroenzymopathy.

Subjects: Blood samples from 373 unrelated asymptomatic adult males who had been exposed to toxic chemicals in different regions (27 cities) of São Paulo State, Brazil, and from 21 unrelated male children suspected of having erythroenzymopathy who were treated at hospitals in São Paulo city were screened for G6PD activity. This study was aproved by the Ethical Commitee of the Faculty of Pharmaceutical Sciences (protocol no. 214/2003) of the University of São Paulo, and formal consent was obtained from all participants prior to enrollment in the study.

Biochemical analysis: Red cell G6PD activity was assayed according to Beutler (1990). Individuals with G6PD activity lower than 70% of normal values were screened for *G6PD* gene mutations by PCR-RFLP (restriction fragment length polymorphism) or PCR-SSCP (single strand conformation polymorphism).

Fifteen of the 373 blood samples (4%) from adult males were G6PD deficient and screened for mutations. One sample with 64.5% of normal G6PD activity (7.8 IU.gHb^-1^.min^-1^ at 37 °C) compared to control levels (12.1 IU.gHb^-1^.min^-1^ at 37 °C) was also included in the mutation study since this value was considered to be borderline for normal activity (according to WHO criteria, normal activity is > 60% of control levels). Twelve of the 21 children were G6PD deficient and were also screened for mutations.

DNA analysis: Genomic DNA was extracted from leukocytes (Salazar *et al.*, 1998) and RFLP analysis for *G6PD* A+376G, *G6PD* A-202A/376G and G6PD Mediterranean 563C polymorphic mutations was done using a procedure modified from Saad *et al.* (1997). The polymerase chain reaction (PCR) for RFLP or SSCP was done in a total volume of 50 μL using 100 ng of genomic DNA, 15 pmol of each oligonucleotide, 0.2 mM of each dNTP, 1 U of DNA polymerase (Biotools), and 1x reaction buffer (10x PCR buffer; Biotools). The primers used were previously described by Beutler *et al.* (1989) and Poggi *et al.* (1990). For RFLP analysis, the PCR products of exons 4, 5 and 6 were digested with *NlaIII*, *FokI* and *MboII* restriction endonucleases, respectively. Negative samples for A+376G, A-202A/376G and Mediterranean563C mutations were analyzed by SSCP, according to Beutler *et al.* (1989) and Poggi *et al.* (1990). The PCR products were denatured in 95% formamide, 0.005% bromophenol blue, 20 mM EDTA and 0.005% xylenecyanol in 10 mM Tris-HCl, pH 8.0, at 95 °C for 8 min followed by rapid cooling on ice. Polyacrylamide gel electrophoresis was done at 600 V, 25 mA and 15 W using GeneGel Excel 12.5/24 in the GenePhor System (Pharmacia Biosciences, Uppsala, Sweden for 90 min at two temperatures (5 °C and 15 °C). The gels were stained with silver nitrate (Sambrook *et al.*, 1989). The resulting bands were compared with a normal control and fragments with any alteration were purified (QIAquick PCR purification Kit, Qiagen Inc., Chastworth, CA, USA) and directly sequenced with sense and antisense primers in a model 3100 capillary automatic sequencer (Applied Biosystem, Foster City, CA, USA). For haplotype determination, six intragenic polymorphic sites were analyzed, as described by Rodrigues *et al.* (2002): exon 5, 376A > G, *FokI*; intron 5, 611C > G, *PvuII*; intron 8, 163C > T, *BspHI*; exon 10, 116G > A, *PstI*; exon 11, 1311 C > T, *Bcl I* and intron 11, 93T > C, *NlaIII.* Restriction enzyme digestions were done according to the manufacturer's instructions (New England Biolabs, Ipswich, MA, USA). The polymorphic haplotypes were recorded as (+) or (-) if the restriction site was present or absent, respectively.

PCR-RFLP analysis showed that 12 of the 15 adult males had *G6PD* A-202A/376G mutation (202G > A and 376G > A mutations). All but one of the G6PD-deficient children (11/12) also had this mutation. All of the mutations had the haplotype VIa (+ + - + - +).

Two G6PD-deficient adults and the child without the *G6PD* A-202A/376G mutation had a different SSCP pattern for exon 8 compared to the controls. This exon was subsequently sequenced and the variant identified as *G6PD* Seattle (844G > C). These variants had the haplotype I (- - + + - -).

The sample with 64.5% G6PD activity showed no mutations in RFLP analysis, but SSCP analysis revealed a different profile in exon 7 compared to the normal control (Figure 1). DNA sequencing of the PCR product of exon 7 revealed a novel point mutation, c.660C > G (Figure 2), that led to the replacement of isoleucin by methionine (I220M). This mutation was associated with haplotype I (- - + + - -) and the new variant was designated as *G6PD São Paulo*.

Polymorphic variants: Most of the individuals included in the DNA analysis had a G6PD A- 202G > A mutation that is frequently found in African descendents from Central Africa, thus confirming the results obtained by Saad *et al.* (1997). In their study of 150 G6PD-deficient blood donors, these authors observed the A- 202G > A mutation in 146 individuals (97.3%), indicating that this variant is the most frequent G6PD deficiency in the population of southeastern Brazil. G6PD Mediterranean but did not detect any G6PD Seattle. In contrast, in a sample from the same population, we found G6PD Seattle but not G6PD Mediterranean. This variation may reflect the ethnic heterogeneity of the population in the state of São Paulo.

Haplotype analysis: All individuals with *G6PD* A- 202G > A had the characteristic haplotype VIa (+ + - + - +), which is very common in Brazilian (Saad *et al.*, 1997) and Portuguese (Rodrigues *et al.*, 2002) populations. Seattle variant carriers had the haplotype I (- - + + - -) that was also present in the G6PD *São Paulo* variant described here.

G6PD São Paulo variant: The large number of G6PD mutations that cause low enzymatic activity (classes I, II and III) indicates that the tertiary structure of the protein probably has many more critical sites that affect the enzymes stability and activity than non-critical sites. However, this conclusion may reflect the fact that most molecular studies of G6PD have been done only in patients with hemolytic crises. Class IV variants have rarely been detected because carriers have near normal G6PD activity, and only five of them have been studied molecularly (Weimer *et al.*, 1993; Takizawa *et al.*, 1987; Beutler and Vulliamy, 2002; Vaca *et al.*, 2003; Sukumar *et al.*, 2003). As shown here we have identified a novel class IV G6PD variant in a male with borderline G6PD activity. A novel mutation c.660 (C > G) was detected in exon 7, surprisingly close to the dimer interface. According to Naylor *et al.* (1996) and Au *et al.* (2000), exon 10, the first half of exon 11, and the second half of exon 6 up to the first half of exon 7 are involved in the dimer interface and are crucial for G6PD stability and activity.

All of the mutations described so far in this region (Harilaou 648T > G, class I; Radlowo 679C > T, class I; “Mexico City” 680G > A, class III; A^-^ 680G > T, class III) significantly reduce G6PD activity (Hirono and Beutler, 1988; Poggi *et al.*, 1990; Beutler *et al.*, 1992; Jablonska-Skwiecinska *et al.*, 1999). In the Harilaou, Radlowo and “Mexico City” A^-^ mutations, the changes occurred between residues with distinct characteristics: 216Phe (nonpolar aromatic) > Leu (nonpolar aliphatic), 227Arg (polar alkaline) > Trp (apolar aromatic), 227Arg (polar alkaline) > Gln (polar neutral), and Arg (polar alkaline) > Leu (apolar aliphatic), respectively (Hirono and Beutler, 1988; Poggi *et al.*, 1990; Beutler *et al.*, 1992; Jablonska-Skwiecinska *et al.*, 1999). It is possible that the substitution of a nonpolar aliphatic amino acid for another amino acid with similar properties (I220L) was crucial for maintaining fairly normal catalytic activity in the novel G6PD variant identified here.

**Figure 1:**
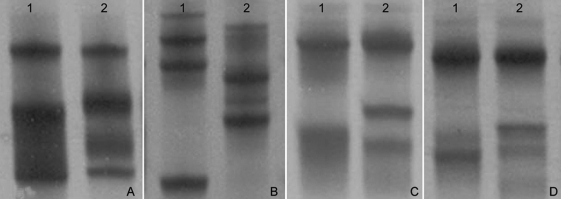
SSCP patterns of *G6PD* exon 7. Electrophoresis was done at 5 °C (A), 8 °C (B), 12 °C (C) and 15 °C (D). Lane 1: G6PD B control. Lane 2: G6PD São Paulo variant.

**Figure 2:**
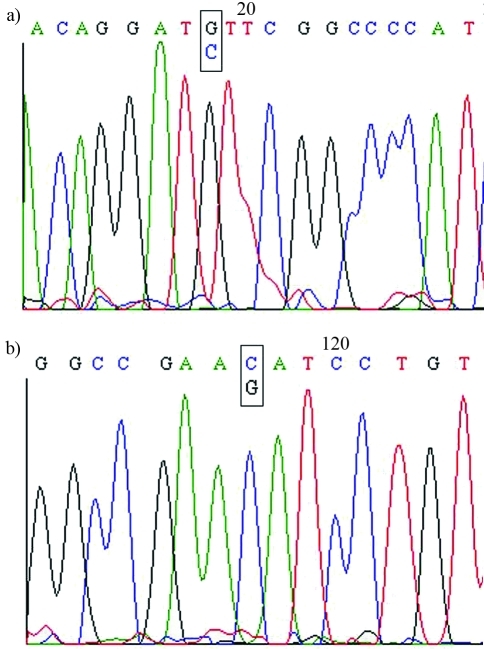
DNA sequencing of *G6PD* exon 7. The use of sense (A) and antisense (B) primers revealed the novel mutation c.660C > G of the *G6PD* São Paulo variant.
